# Defective removal of ribonucleotides from DNA promotes systemic lupus erythematosus

**DOI:** 10.1186/1546-0096-13-S1-O86

**Published:** 2015-09-28

**Authors:** C Günther, B Kind, MAM Reijns, N Berndt, M Martinez-Bueno, C Wolf, V Tüngler, O Chara, YA Lee, N Hübner, YA Lee, L Bicknell, S Blum, C Krug, F Schmidt, C Krug, S Kretschmer, S Koss, KR Astell, G Ramantani, A Bauerfeind, DL Morris, DS Cunninghame Graham, D Bubeck, A Leitch, SH Ralston, EA Blackburn, M Gahr, T Witte, TJ Vyse, I Melchers, E Mangold, MM Nöthen, M Aringer, A Kuhn, K Lüthke, L Unger, A Bley, A Lorenzi, JD Isaacs, D Alexopoulou, K Conrad, A Dahl, A Roers, ME Alarcon-Riquelme, AP Jackson, MA Lee-Kirsch

**Affiliations:** 1 University Hospital Dresden, Derpartment of Dermatology, Dresden, Germany; 2 University Hospital Dresden, Department of Pediatrics, Dresden, Germany; 3 MRC Institute of Genetics and Molecular Medicine, Medical Research Council Human Genetics Unit, Edinburgh, UK; 4 Pfizer-Universidad de Granada-Junta de Andalucía (GENYO), Centro de Genómica e Investigación Oncológica, Granada, Spain; 5 Technical University Dresden, Center for Information Services and High Performance Computing, Dresden, Germany; 6 Max Delbrück Centre for Molecular Medicine, Buch, Berlin, Germany; 7 University of Freiburg, Epilepsy Center, Freiburg, Germany; 8 King's College London, Genetics & Molecular Medicine, London, UK; 9 Imperial College London, Department of Life Sciences, London, UK; 10 MRC Institute of Genetics and Molecular Medicine, University of Edinburgh, Rheumatic Diseases Unit, Edinburgh, UK; 11 Centre for Translational and Chemical Biology, School of Biological Sciences, University of Edinburgh, Edinburgh, UK; 12 Hannover Medical School, Hannover, Germany; 13 University Medical Center, Clinical Research Unit for Rheumatology, Freiburg, Germany; 14 University of Bonn, Institute of Human Genetics, Bonn, Germany; 15 Life & Brain Center, Department of Genomics, Bonn, Germany; 16 University Hospital Dresden, Rheumatology, Department of Internal Medicine III, Dresden, Germany; 17 University of Münster, Department of Dermatology, Münster, Germany; 18 Schwerpunktpraxis Rheumatologie, Dresden, Germany; 19 Städtisches Klinikum Dresden-Friedrichstadt, Dresden, Germany; 20 University of Hamburg, Department of Pediatrics, Hamburg, Germany; 21 Newcastle University, Institute of Cellular Medicine, Newcastle-upon-Tyne, UK; 22 Technical University Dresden, Center for Regenerative Therapies Dresden, Dresden, Germany; 23 Technical University Dresden, Institute for Immunology, Dresden, Germany; 24 Oklahoma Medical Research Foundation, Arthritis and Clinical Immunology Program, Oklahoma City, OK, USA

## 

Systemic lupus erythematosus (SLE) is a multifactorial autoimmune disease in which environmental exposures like virus infection and UV-irradiation trigger activation of the innate and adaptive immune system in genetically predisposed individuals. Heterozygous mutations of the 3' repair exonuclease 1 (TREX1) are associated with SLE. Biallelic mutations in TREX1 and the three subunits of ribonuclease H2 (RNASEH2A-C) cause Aicardi-Goutières syndrome, an inflammatory encephalopathy with clinical overlap with SLE. We therefore investigated the role of RNase H2 in SLE pathogenesis. RNase H2 is responsible for the removal of misincorporated ribonucleotides from DNA and is indispensable for genome integrity. We demonstrated a genetic association for rare RNase H2 sequence variants with SLE. RNase H2-deficient fibroblasts of AGS and SLE patients accumulated ribonucleotides in genomic DNA resulting in chronic low-level DNA damage, constitutive p53 phosphorylation and senescence. Patient fibroblasts proliferated slower than fibroblasts from healthy individuals and showed impairment of cell cycle progression. In addition, patient fibroblasts exhibited constitutive up-regulation of interferon-stimulated genes and an enhanced type I interferon response to the nucleic acid poly(I:C) and UV-irradiation. UV-irradiation induced enhanced cyclobutane pyrimidine dimer formation in ribonucleotide-containing DNA. This suggests that innate immune activation may be caused by immune recognition of DNA metabolites of DNA damage repair and may also explain photosensitivity in SLE patients with RNase H2 mutation. In summary, our findings implicate RNase H2 in the pathogenesis of SLE, and suggest a role of DNA damage-associated pathways in the initiation of autoimmunity.

